# Heme oxygenase-1 nuclear translocation regulates bortezomib-induced cytotoxicity and mediates genomic instability in myeloma cells

**DOI:** 10.18632/oncotarget.7563

**Published:** 2016-02-22

**Authors:** Daniele Tibullo, Ignazio Barbagallo, Cesarina Giallongo, Luca Vanella, Concetta Conticello, Alessandra Romano, Salvatore Saccone, Justyna Godos, Francesco Di Raimondo, Giovanni Li Volti

**Affiliations:** ^1^ Division of Haematology, AOU &ldquo;Policlinico - Vittorio Emanuele&rdquo;, University of Catania, Catania, Italy; ^2^ Department of Drug Sciences, University of Catania, Catania, Italy; ^3^ Department of Biological, Geological and Environmental Sciences, University of Catania, Catania, Italy; ^4^ Department of Biomedical and Biotechnological Sciences, University of Catania, Catania, Italy; ^5^ EuroMediterranean Institute of Science and Technology, Palermo, Italy

**Keywords:** heme oxygenase, oxidative stress, multiple myeloma, endoplasmic reticulum stress, genomic instability

## Abstract

Multiple myeloma (MM) is a clonal B-cell malignancy characterized by an accumulation of clonal plasma cells in the bone marrow leading to bone destruction and bone marrow failure. Several molecular mechanisms underlie chemoresistance among which heme oxygenase-1 (HO-1) could play a major role. The aim of the present research was to evaluate the impact of HO-1 in MM following bortezomib (BTZ) treatment and how HO-1 is implicated in the mechanisms of chemoresistance. MM cells were treated for 24h with BTZ (15 nM), a boronic acid dipeptide inhibitor of the 26S proteasome used in the treatment of patients with MM as first-line therapy. We evaluated cell viability, reactive oxygen species (ROS) formation, endoplasmic reticulum (ER) stress, HO-1 expression and compartmentalization and cellular genetic instability. Results showed that BTZ significantly reduced cell viability in different MM cell lines and induced ER-stress and ROS formation. Concomitantly, we observed a significant overexpression of both HO-1 gene and protein levels. This effect was abolished by concomitant treatment with 4-phenybutirric acid, a molecular chaperone, which is known to reduce ER-stress. Surprisingly, inhibition of HO activity with SnMP (10&mu;M) failed to increase BTZ sensitivity in MM cells whereas inhibition of HO-1 nuclear translocation by E64d, a cysteine protease inhibitor, increased sensitivity to BTZ and decreased genetic instability as measured by cytokinesis-block micronucleus assay. In conclusion, our data suggest that BTZ sensitivity depends on HO-1 nuclear compartmentalization and not on its enzymatic activity and this finding may represent an important tool to overcome BTZ chemoresistance in MM patients.

## INTRODUCTION

Multiple myeloma (MM) is a clonal B-cell malignancy characterized by an accumulation of clonal plasma cells (PC) in the bone marrow (BM) leading to bone destruction and BM failure. MM encompasses a spectrum of clinical variants ranging from benign Monoclonal Gammopathies of Undetermined Significance (MGUS) and smoldering/indolent MM, to more aggressive, disseminated forms of MM and PC leukemia. Despite recent advances in proteasome inhibitor and immunomodulatory drug-based therapies, MM remains largely incurable. The genetic complexity of myeloma is based on intraclonal heterogeneity at the level of a myeloma-propagating cell. Multiple mutations in different pathways trigger a deregulation of the intrinsic biology of the PC and leading to the features of myeloma. The sequential acquisition of multiple genetic events can lead to disease progression and the development of treatment-resistant disease [1]. As far as concerns the mechanisms of pharmacological resistance, several hypotheses and pathways have been advocated; in particular altered redox balance of cancer cells have been proposed as a possible mechanism of chemoresistance. To this regard, cancer cells exhibit persistent reactive oxygen (ROS) species levels leading to an adaptive stress responses and allowing cancer cells to survive with elevated levels of ROS and preserve cellular viability [2]. This aberrantly activated intracellular ROS-scavenging system could have detrimental effects on anticancer drugs that work through accumulation of ROS to stimulate cytotoxicity and cell death. A part from ROS formation, MM cells are characterized by a very high overall level of protein synthesis due to production of a monoclonal immunoglobulin [3] leading to endoplasmic reticulum (ER) stress and are therefore dependent on the unfolded protein response (UPR) for maintenance of protein homeostasis [4, 5]. As long as oxidative stress occurs, cell triggers a complex series of biochemical cascades leading to the upregulation of antioxidant systems in the attempt to maintain the cellular redox balance. Among these various mechanisms, the heme oxygenase (HO) system is emerging as an important regulator of cancer cell redox balance [6, 7].

Heme oxygenase (HO)-1 is an evolutionarily conserved enzyme expressed in mammalian cells. HO-1 is the first and rate-limiting enzyme in heme catabolism, degrading heme to equimolar quantities of carbon monoxide (CO), free iron and biliverdin; biliverdin later converted to bilirubin; free iron is directly sequestered by ferritin [8]. HO-1 is expressed at low levels under basal conditions and it is induced by polyphenols [9–11] and a variety of stimuli such as inflammation, oxidative stress, hyperoxia, hypoxia and trauma [7, 12, 13]. Such upregulation represents an intrinsic defense mechanism to maintain cellular homeostasis and enhance cell survival [14]. In cancer cells, HO-1 is considered to play a major role as an essential survival factor, protecting against chemotherapy-induced increase in ROS [3, 15–19]. The HO-1 protein is anchored to the ER through a transmembrane segment located at the C-terminus [20]. Recently, our research group and others have shown that HO-1 possesses non-enzymatic function related to its ability for protein-protein interaction and/or to translocate in various subcellular compartments. In particular, nuclear localization of HO-1 has been demonstrated in different experimental conditions [6, 21, 22] and may serve to upregulate cytoprotective genes against oxidative stress [23]. We have shown that the protective effect of HO-1 on drug-induced cytotoxicity in leukemic cells does not involve its enzymatic byproducts, but rather its nuclear translocation following proteolytic cleavage [6]. Several lines of evidence suggest that nuclear HO-1 is implicated also as a regulator of DNA repair activities implicated in carcinogenesis [24] and tumor progression [25, 26].

The aim of the present research was to evaluate the impact of HO-1 in MM cells following bortezomib (BTZ) treatment and how HO-1 is implicated in the mechanisms of chemoresistance. Our data suggest that BTZ induces HO-1 via the ER stress pathway and its enzymatic activity is not involved in the mechanisms of chemoresistance. Finally, we showed that nuclear HO-1 confers resistance to BTZ and contributes to genomic instability in MM cells.

## RESULTS

### Effect of bortezomib treatment on cell viability, HO-1 expression and ROS formation

**Figure 1:**
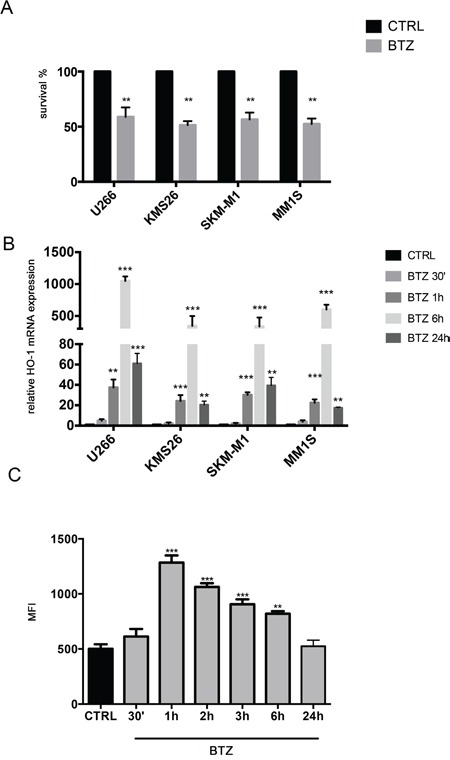
**A.** Cell viability following BTZ (15 nM for 24h) treatment in different multiple myeloma cell lines (**p<0.001 vs untreated control). Results are presented as percentage of the control; **B.** HMOX-1 gene expression following BTZ (15 nM) treatment at different time points and in different multiple myeloma cell lines (**p<0.001 and ***p<0.0001 vs untreated control). Results are expressed as relative expression level (2^−ΔΔCt^); **C.** Reactive Oxygen Species formation following BTZ (15 nM) treatment at different time points and in U266 cell line (**p<0.001 and ***p<0.0001 vs untreated control). Results are expressed as median fluorescence intensity (**p<0.001 and ***p<0.0001 vs untreated control). All values are mean ± SE of four experiments in duplicate.

In the first set of experiments, we exposed 4 different human MM cell lines to the proteosomal inhibitor BTZ. As shown in Figure 1A, all MM cells were sensitive to the drug. We also investigated HO-1 expression in MM cells; all analyzed cell lines over-expressed HO-1 after BTZ treatment with a peak of up-regulation at 6h (Figure 1B) (p<0.0001).

### Bortezomib induces HO-1 expression via ER stress

**Figure 2:**
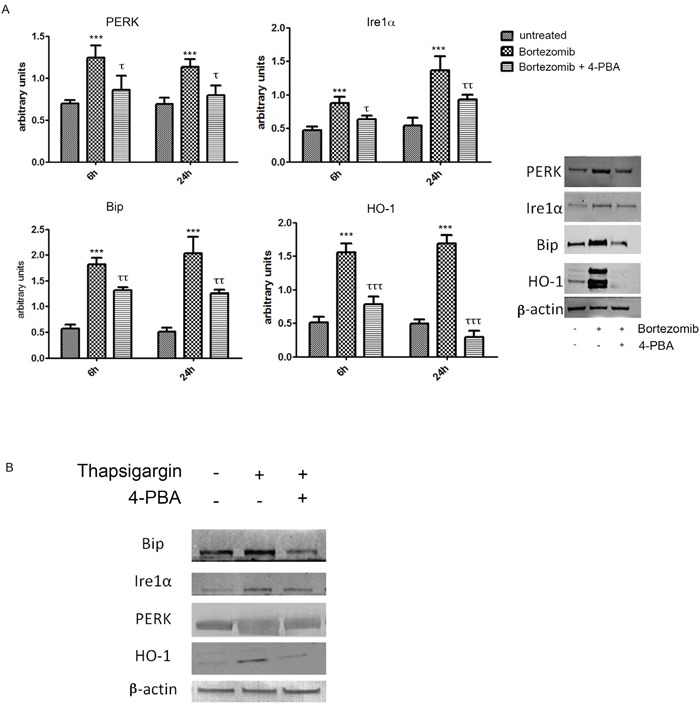
PERK, IRE1α, BiP and HO-1 protein levels in U266 cell cultures treated with BTZ (15 nM for 24h) **A.** or thapsigargin **B.** treatments were visualized by immunoblotting with specific antibodies. Densitometric analysis was performed after normalization with actin. Blots shown are representative of Western blot analysis from four separate experiments. (***p<0.0001 vs untreated control; τ p<0.05 vs BTZ alone; ττ p<0.01 vs BTZ alone). All values are mean ± SE of four experiments in duplicate.

As shown in Figure 2A, BTZ was able to strongly induce expression of all ER stress markers (PERK (PKR-like endoplasmic Reticulum Kinase), molecular chaperone BiP (Binding Immunoglobulin Protein) and IRE1α and also HO-1 (p<0.0001). In order to evaluate whether HO-1 was up regulated via the ER stress pathway, we also treated MM cells with 4-PBA, a chemical chaperone, which served as a negative control. This set of experiments showed that 4-PBA prevented the overexpression of ER-stress related proteins (p<0.05) following BTZ treatment. Interestingly, we also observed a concomitant reduction of HO-1 protein levels (p<0.0001). We further confirmed these results by using thapsigargin, an ER Ca^2+^-ATPase inhibitor [36], served as a positive control. Thapsigargin blocks the ER calcium ATPase pump, leading to the depletion of ER calcium stores [44]. Thapsigargin alone was able to induce all ER stress proteins and HO-1 and this effect was reversed by the addition of 4-PBA (Figure 2B).

### Bortezomib treatment increases nuclear translocation of HO-1

**Figure 3:**
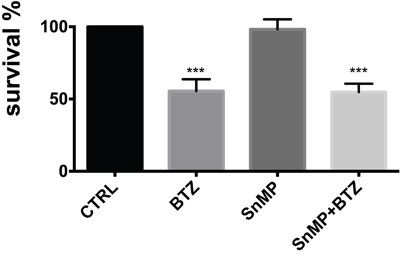
Cell viability following treatment with BTZ (15 nM for 24h) alone or in combination with SnMP (10 μM) Results are presented as percentage of the control. (***p<0.0001 vs untreated control). All values are mean ± SE of four experiments in duplicate.

**Figure 4:**
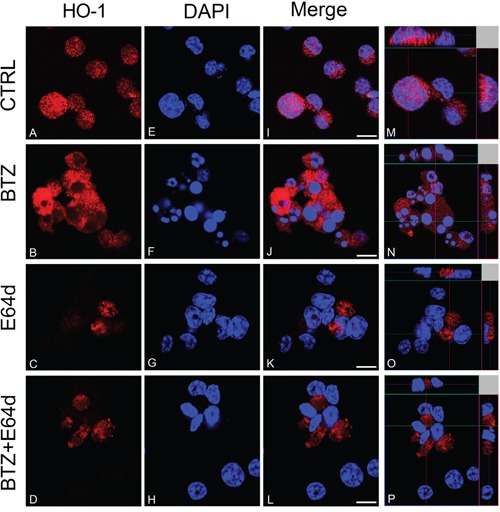
CLSM analysis of HO-1 localization in untreated U266 cell cultures (A, E, I, M) and following BTZ (15 nM for 24h) treatment alone (B, F, J, N) and/or in combination with E64d (20 μM) (C, G, K, O; and D, H, L, P) HO-1 detection was performed by incubation with anti-rabbit monoclonal antibody followed by secondary antibody conjugated to TRITC (red). Counterstaining of cells was performed by using the nuclear dye, DAPI (blue). The photographs result from sequential analysis of the same microscopic field, followed by merging of different images with specific staining. (Scale bars 10μm).

**Figure 5:**
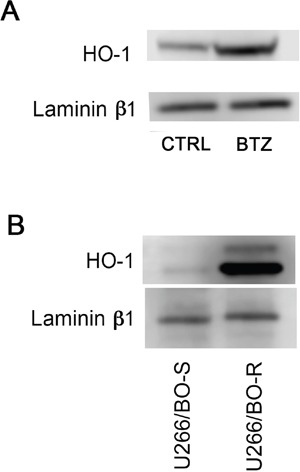
**A.** HO-1 nuclear protein levels in U266 cell cultures treated with BTZ (15 nM for 24h) was visualized by immunoblotting with specific antibodies; **B.** HO-1 nuclear protein levels in U266 sensible (U266/BTZ-S) and resistant (U266/BTZ-R) to BTZ. Laminin b1 shows equal amount of nuclear protein loading in all lanes.

In order to clarify the role of BTZ-induced HO-1, we investigated if its enzymatic activity could influence cytotoxic effect of BTZ in MM cells. Therefore, U266 cells were treated with 10 μM SnMP, a competitive HO inhibitor, either alone or in combination with BTZ. We found that inhibition of enzyme activity did not affect BTZ mediated decrease of cell viability (Figure 3). Furthermore, we investigated HO-1 nuclear localization in U266 cells using both western blot analysis and confocal microscopy. Our data showed that HO-1 is localized into the nucleus as well as in the cytoplasm of untreated cells and BTZ treatment was able to increase nuclear HO-1 levels (Figure 4 and Figure 5A). Our hypothesis suggesting that nuclear HO-1 confers resistance to BTZ is further corroborated by the experiments obtained in a BTZ resistant cell line, which exhibited higher HO-1 expression and nuclear translocation (Figure 5B).

### Inhibition of nuclear translocation of HO-1 increases bortezomib-induced cytotoxicity

**Figure 6:**
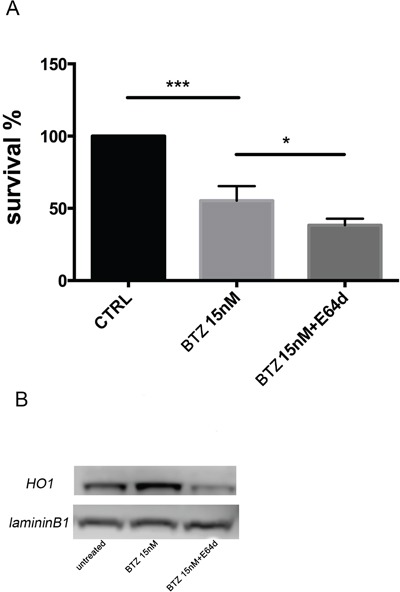
**A.** Cell viability following BTZ (15 nM for 24h) treatment alone or in combination with E64d (20 μM). Results are presented as percentage of the control. (*p<0.05 vs BTZ alone; ***p<0.0001 vs untreated control). All values are mean ± SE of four experiments in duplicate. **B.** HO-1 nuclear protein levels in U266 cell cultures treated with BTZ (15 nM for 24h) treatment alone or in combination with E64d (20 μM). Laminin b1 shows equal amount of nuclear protein loading in all lanes.

To evaluate if nuclear HO-1 translocation could interfere with BTZ response, MM cells were treated with E64d to inhibit the proteolytic cleavage necessary for HO-1 nuclear translocation [6]. Combination of E64d with BTZ potentiated the cytotoxic effect of BTZ (p<0.05; Figure 6A). Western blot and confocal microscopy analysis confirmed the nuclear localization of HO-1 and its reduction after E64d exposure alone and in combination with BTZ (Figure 6B and Figure 4O-4P). These data demonstrated that preventing the accumulation of HO-1 into the nucleus sensitizes U266 cells to BTZ.

### Nuclear HO-1 is involved in genomic instability of MM cells

**Figure 7:**
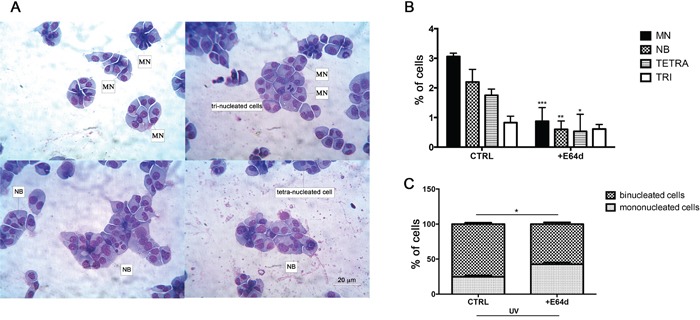
**A.** Representative images showing micronuclei in binucleated cells following Giemsa staining. **B.** Quantification (CBMN assay) of binucleated cells containing micronuclei (MN), nuclear bridge (NB), tetra (TETRA) and trinucleation (TRI). Results were obtained in untreated U266 cell cultures and following E64d (20 μM); **C.** Quantification of binucleated/mononucleated cells (CBPI). Results were obtained in untreated U266 cell cultures and after E64d (20 μM) following exposure to UV light. All values are mean ± SE of four experiments in duplicate. Percentage of cells were calculated over a 1000 counted cells.

MM is characterized by complex genetic abnormalities and aberrant DNA repair pathways that are involved in disease onset and MM progression [25, 37, 38]. To investigate the role of nuclear HO-1 in genetic instability of MM cells, we analyzed U266 cells after treatment with E64d by the cytokinesis-block micronucleus (CBMN) assay. Figure 7A shows representative images of multinucleated cells and binucleated ones with MN and NB. Our data demonstrated that cells treated with E64d exhibited lower percentage of MN (0.87±0.3 vs 3±0.08, p<0.0001), NB (0.6±0.2 vs 2.2±0.3, p<0.001) and tetranucleated cells (0.5±0.4 vs 1.7±0.1, p<0.05) (Figure 7B) when compared with control. In a separate set of experiments we also tested the effect of DNA damage response following UV light exposure. Our data showed that inhibition of nuclear translocation of HO-1 by E64d significantly increased the percentage of mononucleated cells (42.6±2.4 vs 24.6±2, p<0.05) (Figure 7C). In conclusion, these data demonstrated that nuclear HO-1 has a crucial role in genomic instability of MM cells.

## DISCUSSION

Our study showed that BTZ induces HO-1 in MM via the ER stress pathway and that HO-1 nuclear translocation confers resistance to chemotherapy and induces genetic instability in cancer cells. Several lines of evidences substantiate our conclusions. Firstly, we showed that BTZ significantly induces apoptosis, ROS formation and ER stress in different MM cell lines. These data are consistent with previous observations showing that BTZ, a boronic acid dipeptide inhibitor of the 26S proteasome [17] used in the treatment of patients with MM as first-line therapy [39–41], down regulates the expression of several antiapoptotic factors, induces caspase-dependent apoptosis [42–44] and enhances ER stress [45, 46]. MM cells seem to be particularly sensitive to increased ER stress due to the high level of protein synthesis associated with monoclonal component production. It has been shown that proteasome inhibitors induced ER stress, resulting in a UPR that leads to cell death. The mechanisms of cytotoxicity of BTZ against myeloma cells seem to be complex, involving protein translation, DNA damage repair and other pathways [47]. Concomitantly to ROS formation and ER stress we showed a significant induction of HO-1. HO-1 is one of the major ER-associated heme proteins and plays important roles in protection against oxidative and chemical stress. Our data are consistent with previous reports showing that BTZ is able to increase HO-1 expression and thus protecting cells against increased ROS [48]. Under our experimental conditions, HO-1 up-regulation was observed particularly at 6 hours following BTZ treatment on all tested MM cell lines, suggesting a protective role against BTZ-induced ROS. Finally, our data suggest that HO-1 upregulation is dependent on ER-stress since in MM cells treatment with thapsigargin (a specific ER-stress inducer) alone is able to induce HO-1. These effects as well of those related to BTZ on HO-1 were reversed by the treatment with 4-PBA, a chemical chaperone and inhibitor of ER-stress.

It is known that the induction of HO-1 exerts a strong antioxidant and antiapoptotic effect as shown in acute myeloid leukemia resistance to TNFα [49] and in the resistance of pancreatic tumors to gemcitabine [50]. In order to demonstrate the implications of HO-1 in cell resistance to BTZ cytotoxic effect, we co-treated U266 cells with 10 μM SnMP, an inhibitor of HO activity, and BTZ. Our data showed that inhibition of HO enzymatic activity fails to increase BTZ induced apoptosis thus suggesting that HO byproducts (i.e. CO and/or bilirubin/biliverdin) are not involved in the mechanisms of chemoresistance in MM cells. Therefore, on the basis of our previous experiences we further evaluated the intracellular compartmentalization of HO-1 under our experimental conditions. Since its discovery on microsomes, HO-1 localization has been assigned traditionally to the ER. More recently, however, HO-1 has been reported to be present in the nucleus where it regulates gene transcription and cell cycle progression [22, 23, 25]. In this regard, we have previously shown that nuclear HO-1 is able to protect leukemic cells from drug-induced toxicity [6]. Investigating its nuclear localization in MM cells, we found that HO-1 localized also into the nucleus and treatment was able to increase nuclear HO-1 levels. In addition, U266/BTZ-R showed increased levels of nuclear HO-1 compared to their parental cells, suggesting that nuclear protein may be involved in the mechanism of BTZ resistance. Therefore, we next evaluated if nuclear translocation was able to influence drug response by treating MM cells with BTZ in combination with E64d, a cysteine protease inhibitor. Inhibition of the proteolytic cleavage, necessary for HO-1 nuclear translocation, significantly increased the effectiveness of BTZ, showing that HO-1 nuclear translocation could interfere with drug treatment. To this regards, Biswas C. et al [51] showed that nuclear HO-1 modulates the activation of Nrf2, leading to an adaptive reprogramming that enhances antioxidant defenses. This hypothesis is consistent with our data showing that inhibition of nuclear translocation *per se* is not toxic for MM cells but enhances BTZ efficacy.

Models of MM development involve the transformation of a normal PC through a series of related precursor stages, including MGUS and smoldering MM [52, 53]. Furthermore, MM is also recognized to be heterogeneous with distinct molecular subgroups and clonal plasma cells are characterized by the presence of recurrent chromosomal aberrations, reflecting their chromosomal instability [54]. Recently, it has been reported that nuclear HO-1 could be implicated as a regulator of DNA repair activities [25]. Using CBMN assay as technique to measure chromosomal instability, we found that preventing HO-1 nuclear translocation by E64d significantly decreased the percentage of MN, NB and tetranucleated cells. Furthermore, we observed by CBPI assay that E64d treatment improved the G2/M cell cycle control after UV-type DNA damage. All these data demonstrate for the first time that nuclear HO-1 has a crucial role in genomic instability of MM cells. BTZ is able to block DNA repair explaining why a phase II study has shown that the combination of the drug together with high-dose melphalan before autologous stem cell transplantation could increase by 3-fold the complete remission rate [38, 55]. Whether nuclear HO-1 can regulate the transcription of genes implicated in drug-resistance and genomic instability awaits further investigation. As HO-1 does not contain the DNA-binding domain, identification of its interacting proteins in the nucleus may provide a molecular basis underlying its pro-tumorigenic effect in MM.

In conclusion, our data suggest that intracellular HO-1 compartmentalization rather that enzymatic activity is involved in BTZ mediated chemoresistance thus providing an important tool to improve the clinical outcome of MM patients resistant to BTZ.

## MATERIALS AND METHODS

### Cell cultures and treatments

MM cell lines U266, KMS26, MM1S and SKM-M1 were maintained in suspension with RPMI1640 supplemented with 10% FBS and 1% penicillin/streptomycin at 37°C and 5% CO_2_. BTZ resistant cell line (U266/BTZ-R) was obtained alternating exposures first to 10 nM of BTZ and drug-free culture medium for several weeks. To examine the response to BTZ in U266/BTZ-R, we performed experiments after that resistant cell line was regrown in drug-free medium for 3 days (Supplementary Figure 1).

Based on the previous literature data, 15 nM BTZ (Takeda, Rome, Italy) was used in all experiments [27]. For estimation of the effect of BTZ on ER stress markers and HO-1 expression, U266 cells were seeded in 6-well culture plate at density 5×10^5^ cell per well (about 60% confluency), and treated with BTZ alone and in combination with 5 mM 4-Sodium phenylbutyrate (4-PBA, Sigma-Aldrich, Milan, Italy) for 6 and 24h; and with 10 μM thapsigargin (Santa Cruz Biotechnology) alone and in combination with 5 mM 4-PBA for 24h. For viability assay, U266 cells were seeded on 96-well black culture plate (Eppendorf, Milan, Italy) at density 1×10^4^ cell per well, and subsequently treated with 15 nM BTZ alone and in combination with 10 μM Tin-mesoporphyrin IX dichloride (SnMP, Frontier Scientific, Logan, Utah) or 20 μM cysteine protease inhibitor (E64d, Santa Cruz Biotechnology, Santa Cruz, CA, USA) for 24 hours. To investigate translocation of HO-1, U266 cells were seeded directly on Nunc^®^ Lab-Tek^®^ II chamber slides (Sigma-Aldrich, Milan, Italy) and treated with BTZ alone and in combination with 20 μM E64d for 24 hours. All agents were diluted directly in cell culture medium.

### Cell viability assay

Cell viability was assessed using ATPlite 1step assay (PerkinElmer, Milan, Italy) according to the manufacturers’ protocol. Briefly, the 96-well black culture plate was taken from the incubator and equilibrated at room temperature for 30 minutes. Subsequently, to each well containing 100 μl of the cell suspension (5×10^5^ cells/ml), 100 μl of reconstituted reagent was added and the plate was shaken for 20 minutes at 700 rpm using orbital shaker (Stuart Scientific, Staffordshire, UK). The luminescence was measured using Victor3 (PerkinElmer, Milan, Italy). Viability of the cells was expressed as percentage of vitality of untreated cells.

### Determination of intracellular ROS generation

To determine the intracellular ROS generation (mainly superoxide), cells were stained with 5 mM dihydroethidium (DHE, Sigma-Aldrich, Milan, Italy) in PBS for 30 minutes at 37°C. Next, the fluorescence (excitation at 488 nm, emission at 620 nm) was determined by fluorescence-activated cell sorting (FACS, FC500, Beckman Coulter, Milan, Italy) [28].

### Gene expression analysis by real-time PCR (qRT-PCR)

RNA was extracted by Trizol reagent (Invitrogen, Carlsbad, CA, USA). First strand cDNA was then synthesized with Applied Biosystem (Foster City, CA, USA) reverse transcription reagent [29]. HO-1 mRNA expression was assessed by TaqMan Gene Expression, Applied Biosystem and quantified using a fluorescence-based real-time detection method by 7900HT Fast Real Time PCR System (Life technologies, Carlsbad, CA, USA). For each sample, the relative expression level of HO-1 (Hs01110250_m1) mRNA was normalized using GAPDH (Hs02758991_g1) as an invariant control.

### Western blot analysis

Briefly, for western blot analysis 30 μg of protein was loaded onto a 12% polyacrylamide gel Mini-PROTEAN^®^ TGX™ (BIO-RAD, Milan, Italy) followed by electrotransfer to nitrocellulose membrane Trans-Blot^®^ Turbo™ (BIO-RAD, Milan, Italy) using Trans-Blot^®^ SE Semi-Dry Transfer Cell (BIO-RAD, Milan, Italy). Subsequently, membrane was blocked in Odyssey Blocking Buffer (Licor, Milan, Italy) for 1h at room temperature. After blocking, membrane was three times washed in PBS for 5 minutes and incubated with primary antibodies against HO-1 (1:1000) (anti-rabbit, Cat. No. BML-HC3001-0025, Enzo Life Sciences, Milan, Italy), BiP (1:1000) (anti-rabbit, Cat. No. 3177S, Cell Signaling Technology, Milan, Italy), Iron Responsive Element-1α (IRE1α) (1:1000) (anti-rabbit, Cat. No. 3294S, Cell Signaling Technology, Milan, Italy), PERK (1:1000) (anti-rabbit, Cat. No. 5683S, Cell Signaling Technology, Milan, Italy), β-actin (1:1000) (anti-mouse, Cat. No. 4967S, Cell Signaling Technology, Milan, Italy), and laminin B1 (1:1000) (anti-rabbit, Cat. No. sc-5583, Santa Cruz Biotechnology, Santa Cruz, CA, USA), overnight at 4°C. Next day, membranes were three times washed in PBS for 5 minutes and incubated with Infrared anti-mouse IRDye800CW (1:5000) and anti-rabbit IRDye700CW secondary antibodies (1:5000) in PBS/0.5% Tween-20 for 1h at room temperature. All antibodies were diluted in Odyssey Blocking Buffer. The blots were visualized using Odyssey Infrared Imaging Scanner (Licor, Milan, Italy) and protein levels were quantified by densitometric analysis of antibody responses. Data were normalized to protein levels of β-actin for cytoplasmic fraction and laminin B1 for nuclear fraction.

For extraction of nuclear proteins, *NE-PER™ Nuclear and Cytoplasmic Extraction Reagents* (Life technologies, Milan, Italy) were used following manufacturer's instructions.

### Immunofluorescence

Cells were grown directly on coverslips before immunofluorescence. After washing with phosphate-buffered saline (PBS), cells were fixed in in 4% paraformaldehyde (Sigma-Aldrich, Milan, Italy) for 20 minutes at room temperature. After fixation, cells were three times washed in PBS for 5 minutes and blocked in Odyssey Blocking Buffer for 1h at room temperature. Subsequently, the cells were incubated with primary antibody against HO-1 (anti-rabbit, Cat. No. BML-HC3001-0025, Enzo Life Sciences, Milan, Italy) at dilution 1:200 and against β-actin (anti-mouse, Cat. No. 4967S, Cell Signaling Technology, Milan, Italy) at dilution 1:200, overnight at 4°C. Next day, cells were three times washed in PBS for 5 minutes and incubated with secondary antibodies: TRITC (anti-mouse, Cat. No. sc-3796, Santa Cruz Biotechnology,) at dilution 1:200, and FITC (anti-rabbit, Cat. No. sc-2012, Santa Cruz Biotechnology, Santa Cruz, CA, USA) at dilution 1:200 for 1h at room temperature. All antibodies were diluted in Odyssey Blocking Buffer. The slides were mounted with medium containing DAPI (4′, 6-diamidino-2-phenylindole, Santa Cruz Biotechnology, Santa Cruz, CA, USA) to visualize nuclei. The fluorescent images were obtained using a Confocal Laser Scanning Microscopy (CLSM, Zeiss LSM700, Milan, Italy).

### The cytokinesis-block micronucleus (CBMN) assay

The CBMN assay is an established method for assessing chromosomal instability measuring chromosome breakage (micronuclei, MN), nucleoplasmic bridges (NB) and nuclear buds (NBUD) in cultured cells [30]. The frequency of MNi, NB and NBUD was measured as described previously [31]. After 48 h of culture w/o E64d, U266 cells were blocked in cytokinesis by the addiction of Cytochalasin B (CytB; 4,5 μg/ml final concentration). 24 h after adding Cyt-B, cells were harvested by cytocentrifugation. Slides were stained with Giemsa solution. For each sample, 1000 binucleated cells were scored for abnormalities following the criteria specified by Fenech et al [32, 33].

### The cytokinesis block proliferation index (CBPI) assay

Myeloma cell lines were analyzed for regulation of mitotic entry by CBPI assay as described previously [34, 35]. Briefly, cells treated or not with E64d, were exposed to UV-C (2,5 jm^−2^) and subsequently incubated for 72 h with Cyt-B. After cytospin, the cell preparations were stained with Giemsa solution. The cells can be analyzed for their mitotic status (mononucleated, binucleated, multinucleated). Every analyzed condition was scored for the percentage of binucleated/mononucleated cells. Furthermore, to determine whether HO-1 could also influence the cellular response to DNA damage, following the CBPI assay, MM cells were treated for 24 h with E64d, exposed to UV and then incubated with CytB for 72 h. CytB allows the accumulation of dividing cells at the binucleated stage by inhibiting the rate of actin polymerization and the interaction of actin filaments. Therefore, these cells progressed through the G2/M checkpoint and mitosis but fail to divide due to presence of CytB [35]. Under physiological conditions, DNA damage leads cells activate the G2/M cell cycle checkpoint thus increasing the percentage of mononucleated cells in respect of the binucleated.

### Statistical analysis

Data are expressed as mean ± standard error (SE). Significance was assessed by Mann–Whitney U test, one way ANOVA Tukey's multiple comparisons test was used or Student's t test. p<0.05 was considered to be statistically significant.

## SUPPLEMENTARY FIGURE


