# Supplementary material for: Heme oxygenase-1 nuclear translocation regulates bortezomib-induced cytotoxicity and mediates genomic instability in myeloma cells
Source: Oncotarget. 2016 Feb 22;7(20):28868–80. doi: 10.18632/oncotarget.7563 (PMC5045362; doi:10.18632/oncotarget.7563)
Supplement: Supplementary file 1 [file oncotarget-07-28868-s001.pdf]

Heme oxygenase-1 nuclear translocation regulates bortezomib-induced cytotoxicity and mediates genomic instability in myeloma cells

SUPPLEMENTARY FIGURE

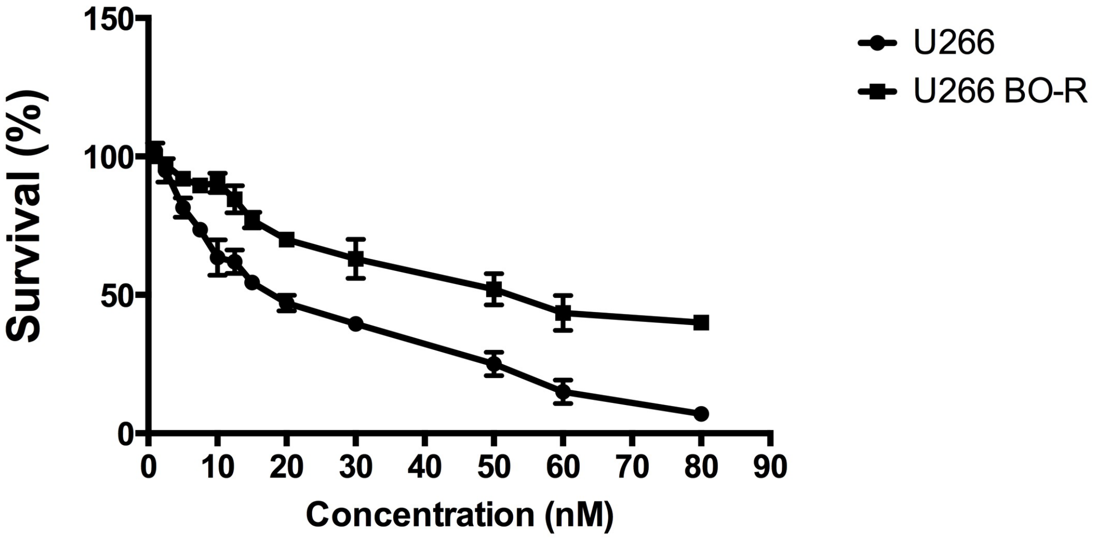

Supplementary Figure S1: Determination of BTZ IC50 value in U266 sensible (U266-R) and U266 resistant (U266-R) cell lines.
